# Continuous Compressed Sensing for Surface Dynamical Processes with Helium Atom Scattering

**DOI:** 10.1038/srep27776

**Published:** 2016-06-15

**Authors:** Alex Jones, Anton Tamtögl, Irene Calvo-Almazán, Anders Hansen

**Affiliations:** 1 Centre for Mathematical Sciences, University of Cambridge, United Kingdom; 2 Cavendish Laboratory, J. J. Thompson Avenue, Cambridge CB3 0HE, United Kingdom

## Abstract

Compressed Sensing (CS) techniques are used to measure and reconstruct surface dynamical processes with a helium spin-echo spectrometer for the first time. Helium atom scattering is a well established method for examining the surface structure and dynamics of materials at atomic sized resolution and the spin-echo technique opens up the possibility of compressing the data acquisition process. CS methods demonstrating the compressibility of spin-echo spectra are presented for several measurements. Recent developments on structured multilevel sampling that are empirically and theoretically shown to substantially improve upon the state of the art CS techniques are implemented. In addition, wavelet based CS approximations, founded on a new continuous CS approach, are used to construct continuous spectra. In order to measure both surface diffusion and surface phonons, which appear usually on different energy scales, standard CS techniques are not sufficient. However, the new continuous CS wavelet approach allows simultaneous analysis of surface phonons and molecular diffusion while reducing acquisition times substantially. The developed methodology is not exclusive to Helium atom scattering and can also be applied to other scattering frameworks such as neutron spin-echo and Raman spectroscopy.

Helium atom scattering has proven to be an invaluable technique to study the structure and dynamics of a wide variety of surfaces ranging from simple metals to reactive and metastable surfaces^1^,^2^,^3^. Helium Spin-Echo (HeSE) spectrsocopy is a novel technique^4^,^5^ which combines the surface sensitivity and the inert, completely non-destructive nature of He atom scattering with the unprecedented energy resolution of the spin-echo method^6^. HeSE is the ideal tool for studying surface dynamical processes within a time window from sub-pico second up to nanosecond time scales. This involves atoms and molecules diffusing on the surface^7^,^8^, phonon vibrations^9^,^10^, etc. Thanks to the work of Van Hove *et al*.^11^ a theoretical framework exists which describes the dynamics of atoms and molecules through the relation of position and time (**r**, *t*) to momentum and energy transfer (Δ**K**, Δ*E*) as a Fourier pair.

In this paper we present a Compressed Sensing (CS) approach for compressing this measurement process, showing that the time needed to reconstruct HeSE spectra can be reduced by several orders of magnitude compared to standard Discrete Fourier Transform (DFT) reconstruction techniques. CS, pioneered by Candès, Donoho, Tao *et al*.^12^,^13^,^14^,^15^ has long been associated with Nuclear Magnetic Resonance (NMR) based applications such as Magnetic Resonance Imaging^15^,^16^ and NMR Spectroscopy^17^. Recently, compressed sensing has also seen applications focusing on Raman spectroscopy measurements^18^ and in molecular dynamics simulations^19^.

Spin-echo spectroscopy shares clear similarities with these fields, such as Fourier transforms arising naturally in data acquisition, however there are also significant differences. In particular, one of the goals of spin-echo spectroscopy is to determine dynamical processes by monitoring the change of polarisation data. Here we consider the whole process of data processing, from polarisation data measurements to the extraction of the molecular dynamics information. Unlike NMR and many other spectroscopy-based applications, after we have performed compression on the initial Fourier transform step we cannot directly use the output data, it must instead undergo several further transforms. Crucially, this includes a non-linear change of variables to momentum/energy space (Δ**K**, Δ*E*). This precludes the use of standard DFT-based CS techniques as this transform distorts the (necessarily discrete) set of values that we can solve for.

Instead a new *continuous* CS approach, recently introduced by Adcock, Hansen *et al*.^20^,^21^,^22^, is used to reconstruct a continuous approximation that avoids discretising entirely. With this method one has the freedom of evaluating the reconstructed function at any point they desire while still having the speed-up benefits of compressive sampling. Such an approach could be used to handle other inverse problems that require further transformations after reconstruction. Moreover, as demonstrated in refs 20, 21, 22 the continuous model avoids the so-called wavelet and inverse “crimes” and hence provides superior reconstructions compared to the classical approach.

Figure 1 shows the process of converting sampled polarisation data to the intermediate scattering function (ISF) *I*(Δ*K, t*) alongside the application of this new CS technique. Note that CS reconstructions in red for the wavelength intensity and scattering functions match the true signal with 9% of the data traditionally used to reconstruct such spectra using direct Fourier inversion without compression.

Moreover, in standard NMR experiments the smallest group of data that is taken in one measurement is typically a line (or path) of data points in k-space, with the exception of new realisations such as electron spin echo envelope modulation^23^. However, in HeSE each measurement corresponds to a single point. This gives us an additional degree of freedom in the data acquisition process which makes the application of CS particularly effective, as one can utilize the new approach of structured multilevel sampling in^21^ to its fullest to boost performance. In particular, by also taking the structure of the signal into account when designing the sampling strategy one can outperform the classical compressed sensing results (see refs 24 and 25 for experimental validation) that are dictated by the estimate on the number of samples *m* to be


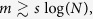


where *s* is the number of non-zero or important coefficients and *N* is the dimension of the vector (The notation 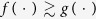 means there is a universal constant *C* > 0 such that *f*(⋅) ≥ *Cg*(⋅)).

However, if a signal has *s* = *M*_1_ + *s*_2_ + … + *s*_*r*_ non-zero coefficients where *M*_1_ denotes the number of the first consecutive non-zero coefficients in the first levels of a wavelet expansion and *s*_*j*_ is the number of non-zero coefficients in the *j*-th level of the wavelet structure then, by using a multilevel sampling procedure^21^, one needs only


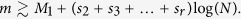


measurements^21^. Typically, the coefficients corresponding to *M*_1_ are the most important, and most of the energy in the signal is contained in these. This is very convenient as we do not have to pay a log factor for these coefficients. In practice this means substantial gain over the standard approaches as demonstrated in recent puplications^24^,^25^.

## Compressing Spin-Echo Spectra

To understand how continuous CS differs from conventional DFT CS we start with a typical 1D Fourier problem where we measure Fourier samples *P* of a wavelength intensity function *ρ* we want to reconstruct:


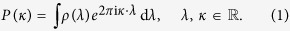


From the above it is immediately recognised that *P* is a Fourier Transform of *ρ* and therefore *ρ* can be obtained by the inverse Fourier transform of *P*. For a general function *ρ* this would require knowing *P*(*κ*) at every point 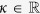 which is unrealistic.

In practice however, the wavelength intensity function *ρ* is treated as a periodic function


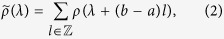


over a fixed interval [*a, b*]. This is convenient because it permits changing the problem to one of handling a Fourier transform to that of handling a Fourier series expansion:


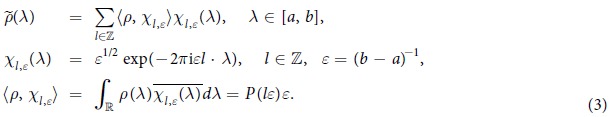


The upshot of (3) is that we now only need the values 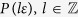, to obtain the wavelength intensity function *ρ*, rather than 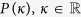.

Typically the next step is to truncate the Fourier series expansion, meaning that one makes the approximation


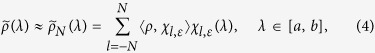


for some fixed 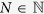. The problem is now feasible as only finitely many data points *l* are required to determine 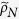.

Up to this point both continuous CS and conventional DFT CS agree. Conventional DFT CS then breaks up the interval [*a, b*] into a uniform grid of *N* points


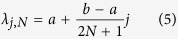


and solves for 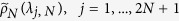. The advantage of doing this is that (4) becomes a vector-matrix equation of the form *g* = *Af* where 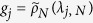, *A* is a DFT matrix and *f* corresponds to samples of *P*. This can be inverted to give *f* = *A*^−1^ *g* where *A*^−1^ is still a DFT matrix, and therefore an isometry, which facilitates the application of CS.

The drawback of this approach is that important information could be lost by only considering 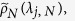 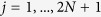  instead of (4). The Fourier series approximation could have been evaluated at any point in the interval [*a, b*] and suddenly the best we can do is reconstruct the 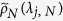, even though we are still working with the same number of Fourier samples. Do we really have to pay this price in order to be able to compress this problem? The answer is: no.

Motivated by the Fourier series (4), one can try approximating *ρ* in terms of a new *Reconstruction Basis* 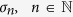



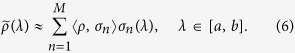


Apart from the benefit of keeping the problem continuous, one also has the freedom to *choose* which basis *σ*_*n*_ to work with, making the approach more versatile than a straight DFT approach. Note that this is similar in spirit to the work on finite rate of innovation^26^ as well as to the concept suggested by Markovich *et al*.^27^.

Since one is still sampling data that corresponds to Fourier coefficients of *ρ*, it is impossible to exclusively work with their choice of basis *σ*_*n*_. Instead one has to convert Fourier series coefficients into coefficients in the basis *σ*_*n*_. This is achieved by working with the infinite change of basis matrix for the two bases:


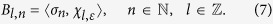


Using this matrix to reconstruct the coefficients 〈*ρ, σ*_*n*_〉, *n* = 1,..., *M* we can then use (6) to approximate 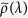. As long as we assume the *σ*_*n*_ form an orthonormal basis then the matrix *B* is an infinite-dimensional isometry which also allows the application of CS thanks to the work of refs 20 and 22.

**Figure 1:**
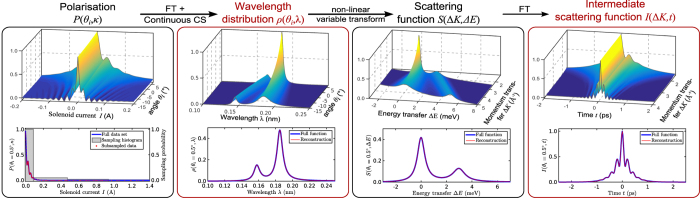
Diagram outlining the various stages of data transformation from measurement in polarisation to the intermediate scattering function (ISF). The upper plots denote the full 2D data while the lower plots are 1D projections/slices with one variable kept constant. The decaying oscillations in the polarisation/ISF are caused by surface phonons. Surface diffusion gives rise to an exponential decay in the polarisation/ISF and a broadening of the peak at Δ*E* = 0 of the scattering function. The stages highlighted in red correspond to the target data we wish to reconstruct. The plotted intensities are in consistent arbitrary units (a.u.). Current spacing/resolution is 2.7 ⋅ 10^−4^A/10233 points and 930 points (≈9%) are subsampled. The experimental variable *κ* is proportional to current *I* according to (9).

This continuous approach has two significant advantages:

- The approximation 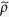 is now a continuous function (as opposed to discrete) that can be evaluated at any point and hence this allows the non-linear change of variables going from the wavelength distribution to the scattering function *S* (as shown in Fig. 1). Such a transform is not possible with conventional discrete CS techniques.
- The approximation 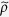 is computed with the actual coefficients in the new expansion of the wavelength function, and hence this approximation has the characteristics of the approximation in the new basis rather than the truncated Fourier series. This means reducing Gibbs ringing and other artefacts coming from Fourier approximations (see refs 20, 21, 22 for details).

Details on the theoretical background of CS, what basis to use, the convex optimisation problems we solve and how to subsample the Fourier data are provided later on in the paper.

### Paper Outline

After a short introduction into HeSE spectroscopy and compressed sensing/continuous compressed sensing we focus on the first Fourier transform step shown in Fig. 1. CS is used to demonstrate the compressibility of phonon detection.

In Surface Diffusion Using Continuous CS, we consider the full cycle of transforms shown in Fig. 1, from polarisation data to the determination of diffusion processes. Thereafter we elaborate on the advantages of the continuous CS technique in order to measure processes that appear on different energy scales.

## Helium Spin-Echo Spectroscopy

The principle of the Helium spin-echo apparatus is the following: A beam of thermal ^3^He is generated from the source in a fixed direction. The nuclear spins are polarised and then rotated by the initial or incoming solenoid before being scattered from the target crystal surface. Afterwards any scattered He atoms heading in the direction of the detector are then rotated by the final or outgoing solenoid and passed through another polarisation filter. Thereby the apparatus achieves an energy resolution of 3 μeV and dynamical processes within a time window spanning from the sub-picosecond regime up to nanoseconds can be observed. While a schematic sketch of the machine used at the Cavendish laboratory can be found in the Supplementary Information further details can be found in Jardine *et al*.^4^.

Key variables that the operator can freely adjust include:

- The currents *I*_*i*_, *I*_*f*_ that run through the initial and final solenoid respectively.
- The scattering geometry, namely the angle of the surface normal relative to the source/detector setup.

The incoming monochromatic He beam can be viewed as a plane wave with propagation wavevector 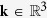 and angular frequency *ω*:


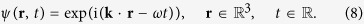


Here, **r** denotes position and *t* is time. The wavevector **k** and wavelength *λ* are related to particle’s momentum **p** by the de Broglie relations via **p** = 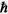
**k** and |**p**| = *p* = 2*π*/*λ*. Furthermore, the frequency *ω* is related to the particle energy *E* by the relation *E* = 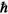
*ω*. Using these relations we can treat **k** as representing momentum and *ω* as energy. Notice that by Formula (8) we have identified two Fourier pairs (**k**, **r**), (*ω, t*).

Upon scattering from a dynamic surface, the wavevector **k** and the energy of the He atom *E* before and after the scattering will typically change. By measuring the probability of a He atom to go from an initial state *i* to a final state *f* information about the surface dynamical processes can be gained. Therefore, we need to measure the properties of the initial and final He beam which is in practice done by changing the current through the solenoids.

In the following we show how the solenoid currents are related to the scattering wavelengths (*λ*_*i*_, *λ*_*f*_). This section follows closely the review of Alexandrowicz and Jardine^5^ and a more detailed description can be found in the Supplementary Information.

### Solenoid Currents and Spin Polarisation

The solenoid currents (*I*_*i*_, *I*_*f*_) and scattering wavelengths (*λ*_*i*_, *λ*_*f*_) share a direct Fourier relationship. Recall that we have two solenoids that generate magnetic fields which rotate the polarisation of the He beam. The solenoid current determines the strength of the magnetic field but it is more convenient to use the experimentally controllable parameter *κ* which is proportional to the current in the solenoids via:


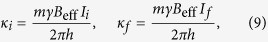


where *γ* is the gyromagnetic ratio of the He atom, *m* is its mass and *B*_eff_ is an apparatus specific constant. The polarisation of the He beam in terms of amplitude and phase can be conveniently written as a complex number. When using the scaled variables of (9) the measured polarisation of the He beam in the detector can be represented as the two-dimensional Fourier transform of *ρ*(*λ*_*i*_, *λ*_*f*_):


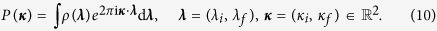


Here *ρ*(*λ*_*i*_, *λ*_*f*_) denotes the *Wavelength Intensity Function* describing the distribution of He atoms that reach the detector according to initial and final wavelengths.

**Figure 2:**
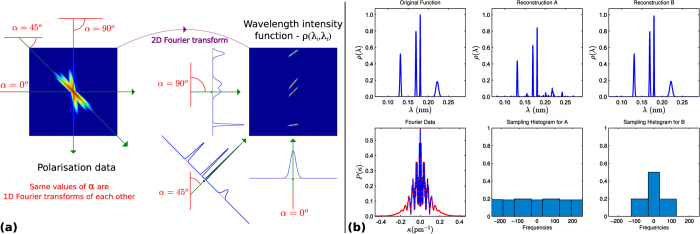
Illustration of a typical wavelength intensity function and the Fourier slice theorem (left panel). The right panel shows a demonstration of the CS for a 1D example shown on the left. (**a**) The wavelength intensity function (on the right) is the two-dimensional Fourier transform of the polarisation data (on the left). The Fourier slice phenomenon is demonstrated by the green lines on the right which indicate the direction of integration with the corresponding projections shown as lines in blue. The green lines on the left hand side represent the one-dimensional Fourier transforms of the projections shown on the right. The polarisation data intensity is shown on a log scale for the sake of readability. (**b**) Demonstrating CS for the 1D 45° projection shown in Fig. 2a, using uniform and multilevel sampling. Samples are taken from the Fourier data according to the sampling histograms shown. (Note that the frequency term in the sampling histogram relates to the values of *κ* at which *P*(*κ*) is sampled). Sampling pattern A is unreliable in reconstructing the rightmost feature as it is the least sparse of the four peaks while sampling pattern B remedies this by taking more of the lower frequency values that it depends upon. Reconstructions are at a resolution of 512 data points.

The change in wavelength can be caused by the creation or annihilation of surface phonons. A short description of the scattering upon surface vibrations can be found in the Supplementary Information. An example of a wavelength intensity function is displayed on the right-hand side of Fig. 2a showing four prominent features. Assuming the features in the plot originate from phonon phenomena on the crystal surface, the classification into creation/annihilation/elastic is according to the energy change Δ*E*^28^:


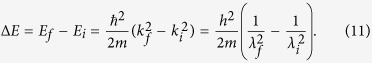


Looking at Fig. 2a as an example, we see that the key features of the wavelength intensity function can be broken down into lines of various slants which suggests that treating the function as one-dimensional would be advantageous. It is precisely because of this decomposition into slanted regions that the two-dimensional problem is often reduced to a one-dimensional one using the Fourier slice theorem: Instead of the two-dimensional (*λ*_*i*_, *λ*_*f*_) space one considers the projection onto a particular line defined by the angle *α*. By restricting the measurement of *P* along the line 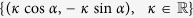 the Fourier transform (10) becomes:


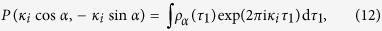


where *ρ*_*α*_(*τ*_1_) denotes the integral of *ρ*(*λ*) along the line 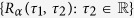. For a more detailed derivation of the Fourier slice theorem please refer to the Supplementary Information.

Figure 2a shows how the Fourier slice theorem applies to the wavelength intensity function. Notice that different angles of integration produce different results, especially when it comes to discerning different features. Since we know beforehand that an elastic peak lies along the line *λ*_*i*_ = *λ*_*f*_ we expect that an integration angle of *α* = *π*/4 will produce the best results for resolving this feature as a single spike.

With this projection, we can treat the problem (12) as a one-dimensional version of (10) with a new wavelength intensity function *ρ*_*α*_(*λ*)


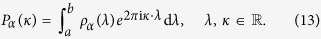


### Compressed Sensing

In this section we shall assume that we have already reduced the problem to one dimension and write *P*_*α*_, *ρ*_*α*_ from (13) as *P, ρ*.

One can discretise (13) by breaking up the interval [*a, b*] into a uniform grid of *N* points *λ*_*j*,*N*_ as in (5), leading to the following matrix equation:


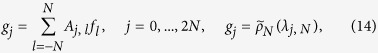


where *f*_*l*_ = Constant(*l*) ⋅ *P*(*lε*) and *A* is a DFT matrix.

Currently, to obtain the full vector (*g*_*j*_)_*j* = 0,..., 2*N*_ we need to know the entire vector (*f*_*l*_)_*l* = −*N*, ..., *N*_. If we only had knowledge of a fraction of the entries of *f* we can no longer use (14) to determine *g* directly as the problem is now underdetermined. Therefore, the problem is not well posed and has to be modified.

The matrix equation (14) can be inverted to give


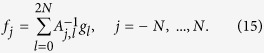


Now suppose that Ω ⊂ {−*N*, ..., *N*} denotes the set of indices corresponding to the samples of *f* that are measured and *P*_Ω_ *f* denotes the projection onto these samples. With this notation *P*_Ω_ *f* denotes the vector of samples that are measured. Therefore when we subsample from {−*N*, ..., *N*} equation (15) becomes


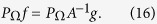


The classical CS approach is to solve this problem via the now well established 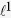 recovery problem


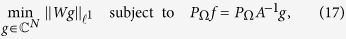


where *W* is some transformation that should make *g* sparse. Typically this is a wavelet transformation. We can then solve this kind of problem quickly and conveniently using convex solvers such as the (SPGL1) algorithm^29^.

The classical idea of CS is that Ω should be chosen uniformly at random. In this case the number of samples *m* = |Ω| must satisfy


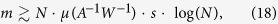


in order to guaranty successful recovery with high probability, where 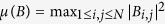. In the case where *B* = *A*^−1^*W*^−1^ as above with any wavelet transform *W* we have that *μ*(*B*) = 1. In this case, as well as many others, uniform random sampling may give suboptimal results and one has to sample with (structured) variable density sampling, see^21^ and references therein. The key problem is that the optimality of variable density sampling depends on the signal itself^21^,^24^,^25^, and thus designing the best sampling pattern is a very delicate task. We will give a short demonstration below.

### How to do structured sampling

The key to understanding structured sampling is to understand the structure of the signal. For example, the coefficients of a signal in a wavelet basis typically have a very specific level structure. This is known as sparsity in levels.

#### Sparsity in levels

Let *x* be a 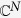 vector. For 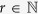 let 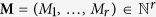 with 1 ≤ *M*_1_ < … < *M*_*r*_ and 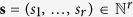, with *s*_*l*_ ≤ *M*_*l*_ − *M*_*l*−1_, *l* = 1,…, *r*, where *M*_0_ = 0. We say that *x* is (**s**, **M**)-sparse if, for each *l* = 1,…, *r*, Δ_*k*_: = supp(*x*) ∩ {*M*_*l*−1_ + 1, …, *M*_*l*_}, satisfies |Δ_*l*_| ≤ *s*_*l*_. This known structure can be utilised when designing the sampling strategy and is the motivation behind multilevel sampling.

#### Multilevel sampling

Let 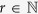, 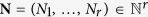 with 1 ≤ *N*_1_ < … < *N*_*r*_, 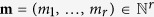, with *m*_*l*_ ≤ *N*_*l*_ − *N*_*l*−1_, *l* = 1, …, *r*, and suppose that Ω_*l*_ ⊆ {*N*_*l*−1_ + 1,…, *N*_*l*_}, |Ω_*l*_| = *m*_*l*_, *l* = 1, …, *r*, are chosen uniformly at random, where *N*_0_ = 0. We refer to the set Ω = Ω_**N**,**m**_ = Ω_1_ ∪ … ∪ Ω_*r*_ as an (**N**, **m**)-multilevel sampling scheme. The key is that in the case of Fourier sampling, represented by the *B* above, combined with a wavelet transform *W* such that the recovery problem becomes (17), the multilevel sampling should match the level structure of the wavelets. More precisely, **N** = **M**. In this case, if *x* is (**s**, **M**)-sparse with total sparsity *s* = *s*_1_ + … + *s*_*r*_ and *s*_1_ = *M*_1_ = *m*_1_, then the total number of samples needed is


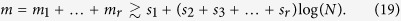


In particular, by utilizing the level structure in the sampling, one can outperform the standard CS results. For a more in-depth analysis and explanation see refs 21 and 25. See also refs 14 and 30 for early versions of this kind of sampling.

### Continuous Compressed Sensing

The motivations behind continuous CS are: (i) to obtain a continuous approximation in the CS reconstruction, as opposed to a discrete approximation, as this allows for an easy non-linear change of variables to obtain the scattering function and the intermediate scattering function. (ii) If an alternative basis to the Fourier representation yields a better representation of the function to be recovered, one wants the freedom to use that. In particular, one can try approximating the wavelength intensity function *ρ* in terms of a new *Reconstruction Basis* 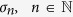:


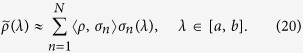


For technical reasons, one often requires these functions to form an orthonormal basis of *L*^2^[*a, b*], e.g. Legendre polynomials, splines, wavelets etc., although this condition can be relaxed to other groups of functions like frames^31^. For this paper we shall be using Daubechies wavelets^32^ exclusively as our reconstruction basis. Let us quickly discuss *why* one would want to work with another basis.

Apart from the benefit of keeping the problem continuous, one also has the freedom to *choose* which basis *σ*_*n*_ to work with, making the approach more versatile than a straight DFT approach, where we are essentially forced to work with a pixel basis every time.

Furthermore, the notion of *sparsity* is now in terms of the coefficients 〈*ρ, σ*_*n*_〉, which means we have the additional advantage of choosing a basis that makes the function *ρ* sparse. As we shall see, this opens up the possibility of using compressed sensing where traditional sparsity does not hold. In addition, this approach is closer to the philosophy that *ρ* being sparse should relate to *ρ* having low information content; a choice of basis *σ*_*n*_ that makes *ρ* sparse tells us how to (approximately) express the function *ρ* with a few non-zero coefficients.

Since one is still sampling data that corresponds to Fourier coefficients of *ρ*, it is impossible to exclusively work with their choice of basis *σ*_*n*_. Instead one has to convert Fourier series coefficients into coefficients in the basis *σ*_*n*_. This is achieved by working with the infinite change of basis matrix for the two bases:


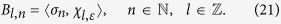


As opposed to (17), we now end up solving the infinite dimensional convex optimisation problem of finding


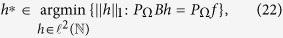


Again *P*_Ω_ denotes the projection onto the samples we have taken. In practice we cannot solve for the infinite solution to (22), therefore we truncate the reconstruction basis in a similar fashion to how we truncate the Fourier basis. This means we end up computing


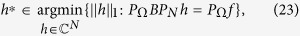


where *P*_*N*_ denotes the projection onto the first *N* functions in the reconstruction basis. This problem is now numerically feasible since the submatrix *P*_Ω_*BP*_*N*_ is now finite (see^21^ for estimates on how to choose *N*). The solution to (23), let’s say h^*^, is recognised as the (approximate) wavelet coefficients of the intensity function. We can then use these wavelet coefficients to compute an approximation to *ρ* evaluated at any point on the interval [*a, b*] by following (20):


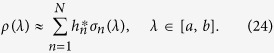


From here one can use the same multilevel sampling techniques as the discrete case to reconstruct (**s**, **M**)-sparse coefficients. Moreover, the sampling rule (19) also applies in this case. Note, however, that solving (23) is very different from solving (17). Indeed, *B* in (21) is an infinite matrix, moreover *P*_Ω_*BP*_*N*_ ≠ *P*_Ω_*A*^−1^*W*^−1^ where we recall *A*^−1^ and *W*^−1^ from (17). Both approaches, (23) and (17), are approximations to the true continuous problem (22), however, the discretisation is done differently. The discretisation in (17) turns out to be suboptimal, which can be seen as follows. As discussed in Compressing Spin-Echo Spectra, if *g* = *Af*, where *f* is a finite vector consisting of the samples of *P* from (1), then *g* is a vector that is a rasterised version of the truncated Fourier series approximation to *ρ* (the true solution we are seeking). Thus, if we apply a discrete wavelet transform *W* to *g*, we see that the result 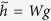 is precisely the vector of the wavelet coefficients of the truncated Fourier series approximation to *ρ*. This means that whatever artefacts the truncated Fourier series approximation suffers from, such as Gibbs ringing, are also transferred to *Wg*. Hence, by solving (17) we will, at best, recover the wavelet coefficients of the truncated Fourier series that may be a suboptimal approximation. The wavelet coefficients of the truncated Fourier series are of little interest. What we want are the wavelet coefficients of the true solution *ρ*. This can be formulated via the infinite linear system 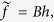 where 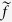 is the infinite vector of all samples of the values *P*(*lε*), 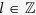, and *h* is the infinite vector of all the true wavelet coefficients of *ρ*. In comparison, the discretisation based on the DFT yields 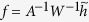, where 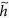 is the vector of wavelet coefficients of the truncated Fourier series. Note that (22) is an infinite-dimensional problem, thus, we need to approximate it via (23). However, as *N* grows, any solution to (23) converges to a solution to (22).

(23) is not based on the traditional DFT, however, it is possible to find a fast *n* log(*n*) implementation for applying the matrix *P*_Ω_*BP*_*N*_, the finite section of the infinite matrix *B*, to any vector. For further explanation and numerical examples demonstrating the benefits of the continuous approach and the differences with the discrete approach, see^20^,^21^,^22^.

## CS for Phonon Detection

In this section we look at the performance of the CS approach described in the previous section by looking at examples of phonon detection. We shall first look at its effects on the one-dimensional projections shown previously and then focus on a real ^3^He spectrum for scattering of gold where more exotic signal behaviour is present.

### Simulated 1D Example

For consistency with previous sections we shall first work with the 45° projection shown earlier. Although this is a simplified model it clearly demonstrates some of the basic properties of compressed sensing. Reconstructions are shown in Fig. 2b.

Recall that we project along a 45° angle in an attempt to reduce the spread caused by the inaccuracy of the wavelength of the incident He^3^ beam. In particular, since we know that there will always be an elastic feature in the wavelength intensity function which itself is slanted at 45°, this choice is seen as ideal for refocusing the various phonon features to be closer to that of a delta spike. Not only is this useful in preventing features from overlapping each other but this also increases sparsity which is ideal for compressed sensing; if features are sparse then by the rule (18) we can subsample to a great degree since the signal itself is very sparse. In this case, a change of basis may not be needed.

If one goes for a uniformly random approach to subsampling (as in Reconstruction A), as opposed to multilevel sampling discussed above, then there is only so far that one can go before problems occur. At around 20% subsampling, the reconstruction becomes unreliable in recovering the least sparse of the features on the far right. With 30% the rightmost feature is typically reconstructed but it is nonetheless unreliable. There is however an even more effective way of reliably reconstructing the rightmost feature by using multilevel sampling (as in Reconstruction B). The theory on how to design optimal structured multilevel sampling strategies is very new^21^,^24^,^25^ and this is a highly unexplored topic. We do not attempt to seek optimality here, as this paper is about establishing the effectiveness of CS in HeSE. Note that, since there is no wavelet change of basis (*W* = *I*, the identity, in (17)) in this case, the theoretical understanding of the effect of multilevel sampling is not fully understood. This is still work in progress together with optimality conditions.

### Real Phonon Spectrum

As we have already mentioned, real phonon spectra contain more exotic features than the simulation given in the previous example. Naturally noise adds to the data due to some experimental uncertainties of the measurement, but more unusual are the relative sizes and shapes of the various features.

**Figure 3:**
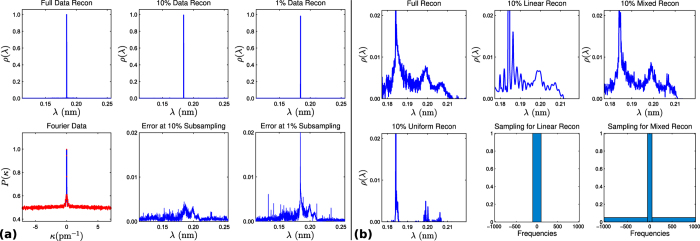
Compressed sensing reconstructions for a measured gold(111) phonon spectrum. (**a**) Compressed sensing reconstructions for a gold phonon spectrum (a.u.): With this choice of viewing range, only the elastic peak can be clearly seen. In this figure the sampling is performed uniformly at random which recovers the highly sparse central peak. This is suboptimal compared to the multilevel sampling in Fig. 3b. Frequencies sampled are from the range {−1024, ..., 1023} and reconstructions are at a resolution of 2048. (**b**) Compressed sensing reconstructions for the same gold phonon spectrum as in Fig. 3a, zoomed in so that features beside the elastic peak are visible. Notice that the smaller features shown here are over 200 times smaller than the elastic peak. Reconstructions shown here are not only uniform (as in the bottom left graph) but also linear (i.e. straight Fourier series) and non-linear examples using roughly the same number of samples across each.

In Fig. 3a we have uniform sampling reconstructions for a typical gold(111) spectrum (for more details see Supplementary Information) with projection at 45° to focus on the elastic peak, which is the only clearly visible feature in the graphs. The peak is extremely fine and is in fact even smaller than the pixel resolution used for reconstruction (2048) which can be determined from the observation that the Fourier data has yet to decay to zero near the highest frequencies. Consequently this is an ideal situation for CS since this feature is almost as sparse as can be. Hence, one can subsample to a much greater degree (e.g. 1%) than in the previous example.

However, what has happened to the other inelastic features in this spectrum? At first one might come to the conclusion that they are not there at all, but, focusing on a small part of this spectrum reveals features that are over 200 times smaller than the measured intensity of the large elastic spike. Figure 3b shows various CS reconstructions zoomed in on this region. Notice that we still have the elastic peak visible, along with a couple of more (and less sparse) features. Like in the previous example, we expect the smooth features to be more dependent upon the lower frequency samples. Therefore, when we attempt to take just the first 10% of samples all from the lowest frequencies (the *linear* reconstruction) we find that these features are at the very least present, unlike the 10% uniform sampling approach where only the central peak remains. On the other hand, the central peak suffers from Gibbs artifacts which manifests themselves as wave like features near the central peak as well as broadening of the peak itself.

Instead, one can opt for a mix of these methods by taking the first 5% of samples from the lowest frequencies and the other 5% taken uniformly from the rest. This approach empirically performs the best out of the three methods, resolving the low resolution features without the Gibbs artefacts of the linear approach. Note that, as demonstrated in^21^,^24^, the optimal sampling procedure is signal structure dependent. How to choose optimal multilevel sampling is beyond the scope of this paper, and we have deliberately chosen a simple two-level sampling pattern, which is a reasonable all rounder, in order to demonstrate the effectiveness of the sampling technique.

### Comparing CS Techniques

In this section we look at an example of how the continuous wavelet approach to compressed sensing can be used to tackle problems that are beyond the capabilities of the traditional compressed sensing approach described earlier.

Prior to data acquisition the spacing in current (equivalent to spacing in *κ*) must be chosen, which in turn determines the length of the wavelength window [*a, b*] that the wavelength intensity function *ρ* is constructed over. If *ρ* is not truly supported on this window, then by (2) we instead reconstruct the periodised version of *ρ*. In particular, if peaks in the intensity function decay particularly slowly relevant to the window then the intensity function will stay considerably above zero throughout that window. Because of this, the traditional compressed sensing approach applied earlier cannot be used successfully here as the function is maximally non-sparse.

However, if one recalls the wavelet reconstruction bases that are used, then one quickly notices that they both have a constant function as the first basis function. This effectively means that the base level caused by slow decay is captured by this single basis function, which keeps the function sparse in these bases.

**Figure 4:**
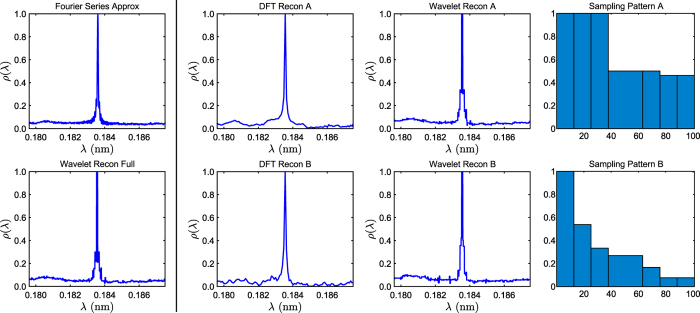
(Continuous vs discrete CS) Reconstructions of a spin-echo spectrum (a.u.) of CoPc molecules deposited on Ag(001) with a noticeable baseline feature. (See Supplementary Information for details on the sample preparation). In the DFT reconstructions the baseline level of around 0.1 is no longer flat leaving bumpy artefacts while the wavelet reconstructions preserve this flat feature. As we are only subsampling from 101 frequency points, considerable Gibbs artefacts are present in the Fourier series approximation. The DFT reconstructions have a resolution of 101 points, while the continuous Fourier series and wavelet reconstructions have been rasterised at a resolution ten times this number.

Figure 4 compares the two compression techniques for the diffusion of cobalt phthalocyanine (CoPc) on Ag(001) with an observable baseline feature. The full set of polarisation data points only corresponds to the first 101 frequencies and therefore there are noticeable Gibbs artefacts around the elastic peak in the Fourier series approximation. This strongly suggests that the Fourier series approximation here is not a particularly accurate approximation to the true intensity function.

Furthermore, the wavelet approximation aims to reconstruct the true underlying continuous wavelength intensity function, unlike the DFT approach which attempts to reconstruct a discretised form of the Fourier series approximation. Consequently, even with full sampling, the wavelet reconstruction is noticeably different to the Fourier series approximation. This reflects the fact that, as we are handling real data, we cannot directly compare to the true underlying wavelength intensity function.

Regardless we clearly observe that the baseline feature is preserved under subsampling using wavelets where the DFT approach clearly fails, matching predictions based on sparsity observations earlier. Note that both techniques use exactly the same samples. While we are able to subsample to a reasonable degree here (≈33%), one should ideally work with a larger range of frequencies to truly exploit the benefits of this approach, i.e. subsampling from polarisation data with thousands of points rather than hundreds.

### Surface Diffusion Using Continuous CS

Surface diffusion, in contrast to phonon studies, deals with species (atoms, molecules) adsorbed on surfaces which diffuse over large distances. It aims to identify the main mechanisms governing the motion on surfaces (i.e. the energetic landscape governing the adsorbate dynamics) and, therefore, to characterize their diffusive behaviour.

**Figure 5:**
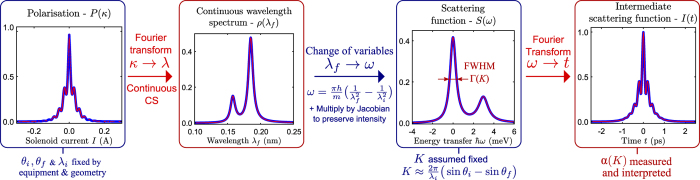
A detailed 1D description of the full loop presented in Fig. 1. Blue lines denote the true underlying signal and red lines denote the reconstructions (except for the left panel where the red dots denote the sampling points). Sampling points are taken according to the sampling histogram present in Fig. 1. Note that there is no comparison with standard discrete CS here as the change of variable technique is impossible in the discrete setup. This can only be done in the continuous case.

The Van Hove formalism of correlation functions in time and space^11^, initially developed for neutron scattering (see Sec. S.5 in the Supplementary Information for more details), provides us with a theoretical formalism to understand and interpret the experimentally determined ISF *I*(**K**, *t*) in terms of diffusive regimes. (Here we have omitted the Δ in the notation of the momentum transfer Δ**K** for the sake of simplicity.) The Van Hove correlation contains all the information related to the dynamics of particles in the system but it cannot be directly measured. However, its properties can be inferred from partial knowledge of the SF/ISF (see Fig. 5). In particular, different diffusive regimes can be identified through the *dephasing rate α*(**K**), which describes the decay in time *t* of the ISF as a function of **K**^33^:


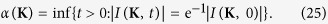


Its Fourier pair is the so-called *quasi-elastic broadening* Γ(**K**) and is defined as the half-width-half-maximum of the quasi-elastic profile in the SF, i.e in the peak centred around Δ*E* = 0 of the SF in Fig. 5.

Therefore, it is essential to determine the “true” ISF from the measured polarisation *P*(*κ*). The processing of the data from *P*(*κ*) to the ISF requires to go through a series of Fourier transformations and change of variables as shown in Fig. 5. The drawback is that the change from wavelength to energy in (11) is incompatible with the standard DFT based CS methods because the uniform grid of coordinates in wavelength space is transformed onto a non-uniform grid in (**K**, Δ*E*)-space (see Sec. S.6 in the Supplementary Information for more details on this variable transform). With continuous CS one can work backwards, first specifying a uniform grid in (**K**, Δ*E*)-space which is converted to a non-uniform grid in wavelength space. As a result, since the reconstructed solution of (22) is a function rather than a vector, one can directly sample the wavelength intensity function on this non-uniform grid and compute the SF on a uniform energy grid. This allows to apply the standard DFT algorithm to obtain the final ISF.

### Measuring Diffusion and Phonons in One Go

Through the different sections of this paper we have seen that the HeSE technique can explore dynamical processes spanning from phonon measurements in the sub-picosecond time window up to diffusion in the cents of picoseconds scale. Yet the continuous CS approach enables us not only to reduce the measurement times, it is also a powerful method to circumvent the experimental complications when trying to capture very different dynamical processes within a single set of data.

Firstly, the capability to reconstruct the spectrum out of a subsampled set of measured currents allows to cover a very large current window with a high resolution in a reasonable measurement time. This provides us with a very broad and highly resolved energy window where we can separate accurately the phonon (the inelastic peak in the spectrum) from the diffusive contribution (the quasi-elastic peak centered at Δ*E* = 0 meV). Secondly, the flexibility of the continuous CS approach allows us to evaluate the reconstructed signal in any desired wavelength grid which greatly simplifies the application of the standard DFT algorithm linking the last two stages of the data processing, from the SF to the ISF in Fig. 5.

Hence the CS approach provides us with a flexible framework where the different physical processes contained in a single data set can be disentangled and analysed in the energy domain (where inelastic features such as phonons can be easily investigated) or in the time domain (where diffusive processes are easier to characterise).

## Conclusion and Summary

We have used compressed sensing (CS) techniques to measure and reconstruct surface dynamical processes with a helium spin-echo spectrometer. This work demonstrates that in particular the continuous CS approach can be used to reduce measurement times by at least an order of magnitude whilst capturing both phonon and diffusion processes simultaneously.

Continuous CS allows us to reconstruct spectra with a very broad and highly resolved energy window in a reasonable measurement time so that we can separate accurately phonon events and diffusion processes measured in a single data set. Since the continuous CS approach allows to evaluate the reconstructed signal on any desired grid this makes it easy to switch between the energy and the time domain and to analyse the underlying physical processes. Not only has this made current helium spin-echo experiments more convenient, but this has also brought forward future projects that were originally deemed too time-consuming to measure. Eventually the final goal is to capture the entire scattering function over all of (**K**, Δ*E*)-space using a two-dimensional continuous CS method.

The developed methodology can also be applied to other scattering frameworks and the authors hope that these advances will be quickly brought to the attention of the neutron scattering and X-ray communities.

## Additional Information

**How to cite this article**: Jones, A. *et al*. Continuous Compressed Sensing for Surface Dynamical Processes with Helium Atom Scattering. *Sci. Rep.* **6**, 27776; doi: 10.1038/srep27776 (2016).

## Supplementary Material

Supplementary Information
