# Supplementary material for: Continuous Compressed Sensing for Surface Dynamical Processes with Helium Atom Scattering
Source: Sci Rep. 2016 Jun 15;6:27776. doi: 10.1038/srep27776 (PMC4908413; doi:10.1038/srep27776)
Supplement: Supplementary Information [file srep27776-s1.pdf]

Continuous Compressed Sensing for Surface Dynamical Processes with Helium Atom Scattering: Supplementary Information

Alex Jones[1,+], **Anton Tamtögl**[2,*,+], **Irene Calvo-Almazán**[2], **and Anders Hansen**[1]

[1]Centre for Mathematical Sciences, University of Cambridge,United Kingdom
[2]Cavendish Laboratory, J. J. Thompson Avenue, Cambridge CB3 0HE, United Kingdom
[*]tamtoegl@gmail.com
[+]these authors contributed equally to this work

April 20, 2016

S.1 Setup of the apparatus

The ^{3}He spin-echo apparatus is based on the same principle as neutron spin-echo spectrometers. However, instead of neutrons which penetrate into the bulk, ^{3}He is used. The repulsive part of the ^{3}He/surface interaction potential prevents the He atoms from penetrating into the surface layer of materials. Consequently they are ideal as a surface scattering probe that allows to truly understand surface processes.[S1]

A schematic drawing of the Cambridge spin-echo apparatus is shown in Fig. S1. An unpolarised ^{3}He beam is created in the source at first. Since ^{3}He exhibits an overall spin of one half a strong magnetic field can be used to polarise the beam. The polarised beam then passes through a solenoid field which gives rise to a precession of the spin around the beam axis before it is scattered from the sample. The scattered beam is again passed through an identical but reversed solenoid field before it passes through an analyser magnet and is finally detected. Since the rate of precession depends on the time that the atom spent in the field and hence the velocity of the atom, the final accumulated precession angle reflects any energy change caused by the scattering at the sample (see S.3).

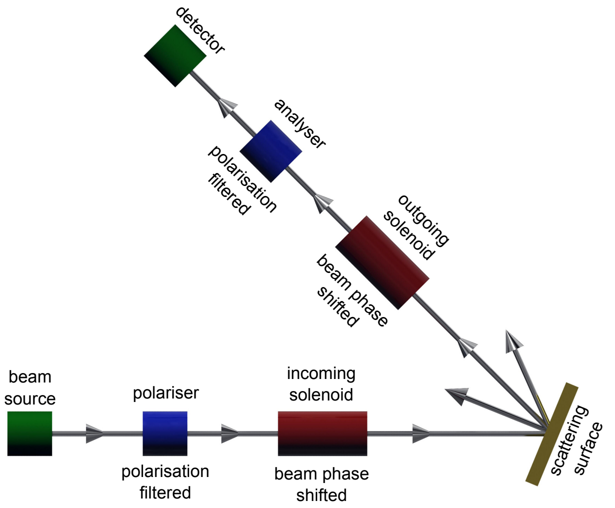

Figure S1. Outline of the Cambridge spin-echo apparatus: An unpolarised beam of thermal ^{3}He is generated from the source in a fixed direction. The nuclear spins are polarised and then rotated by the incoming (initial) solenoid before being scattering upon the target crystal surface. Afterwards any scattered He atoms heading in the direction of the detector are then rotated by the outgoing (final) solenoid and passed through another polarisation filter before being detected.

S.2 Phonons

Detailed descriptions about lattice vibrations in a solid can be found in most solid state physics textbooks.[S2] At the surface the altered environment with respect to the bulk modifies the dynamics to give rise to new vibrational modes. This new vibrational modes are called surface phonons due to their localisation at the surface.[S3–S5]

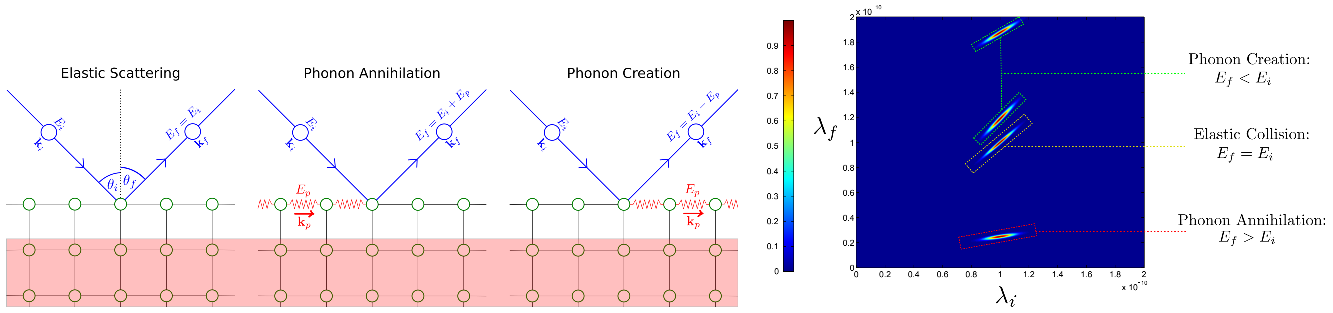

(a) Illustration of three possible scattering events: The ^{3}He atom (in blue) can either be scattered elastically or gain/lose energy via the annihilation/creation of a surface phonon. The fact that the atom cannot penetrate into the bulk is indicated by the red region.

(b) A typical wavelength intensity function with key features highlighted. Classification of the features is deduced from (11).

Figure S2. Illustration of phonon events and an example of a wavelength intensity function

Now we look at how phonons can influence the scattering of the He beam. Fig. S2a shows three possible outcomes for the scattering of a single He atom on a crystal surface. The figure shows a cross section of the crystal where the path of the He beam lies in the same cross section, therefore, we are effectively working with a two dimensional problem. We shall assume there is conservation of energy before and after scattering and that the momentum is conserved parallel to the surface of the crystal (recall that the ^{3}He beam cannot penetrate into the bulk which is why we only consider momentum parallel to the surface). If we let little i, f denote the initial and final He states, little p a possible phonon, $\mathbf{k}$ a wavevector and $k = |\mathbf{k}|$ its magnitude, then by conservation of energy and momentum

$$E_f = E_i + \Delta E, \qquad K_i = K_f + \Delta K, \tag{S1}$$

where $K_i = k_i \sin\theta_i, K_f = k_f \sin\theta_f$ denote the projections of the initial and final momentum $\mathbf{k}_i, \mathbf{k}_f$ onto the surface, provided that the scattering plane defined by $\mathbf{k}_i, \mathbf{k}_f$ contains the surface's normal vector. The change in energy ΔE is equal to the change in kinetic energy of the Helium particle:

$$\Delta E = E_f - E_i = \frac{\hbar^2}{2m}k_f^2 - \frac{\hbar^2}{2m}k_i^2. \tag{S2}$$

The classification into phonon creation/annihilation and elastic scattering originates from (S2). Fig. S2b shows a close-up of the wavelength intensity function presented in the main text, along with possible phonon creation and annihilation events labelled.

In the case of elastic scattering ($\Delta E = 0$) we have $k_i = k_f$ and $\Delta K = G$ where G corresponds to a reciprocal lattice vector consistent with the Laue equations. Suppose now that a He atom annihilates a surface phonon with energy $\Delta E = \hbar\omega_p$. For the momentum transfer $\Delta K = k_p + G$ holds where $\mathbf{k}_p$ is the wavevector of the phonon and $\mathbf{G}$ a surface reciprocal lattice vector and the norm of these vectors can be directly added if $\Delta\mathbf{K}$ is parallel to a high symmetry direction of the crystal.

S.3 From Solenoid Currents to the Spin Phase

The polarisation of the helium spin can be encoded by a phase quantity ϕ. We assume that the relationship between the generated field strength B in the solenoids and the current I flowing through the coil is linear, i.e. $B = B_{\text{eff}} \cdot I$ for some constant B_{eff}. If the solenoid has length L then the total accumulated phase has the form

$$\phi = \frac{\gamma}{V} B_{\text{eff}} I, \tag{S3}$$

where V denotes the velocity of the He atom and γ is the gyromagnetic ratio of the He atom. Therefore if ϕ_i denotes the accumulated phase in the first coil and ϕ_f the phase in the second then

$$\phi = \phi_i + \phi_f = \gamma B_{\text{eff}} \left(\frac{I_i}{V_i} + \frac{I_f}{V_f} \right), \tag{S4}$$

where we assume that the currents I_i, I_f and velocities V_i, V_f are different but the length L and constant B_{eff} is the same. We observe that the incoming velocity V_i is related to the incoming wavelength via the de Broglie relation, i.e. $V_i = p_i m^{-1} = h(m\lambda_i)^{-1}$, where λ_i denotes the wavelength of the beam. Consequently, (S4) becomes

$$\phi = m\gamma B_{\text{eff}} h^{-1}\big(I_i\lambda_i + I_f\lambda_f\big). \tag{S5}$$

Now suppose that polarisation is rotated in the xy-plane and is initially polarised in the x-direction. Then, assuming that the analyser near the detector is also in the x-direction, the signal received has the form[S6]

$$P_{x,I}(I_i, I_f) = \langle\cos\phi\rangle_\rho = \int \rho(\lambda_i,\lambda_f)\cos\Big(m\gamma B_{\text{eff}} h^{-1}\big(I_i\lambda_i + I_f\lambda_f\big)\Big)\,\mathrm{d}\lambda_i\mathrm{d}\lambda_f. \tag{S6}$$

Here $\rho(\lambda_i,\lambda_f)$ denotes the *Wavelength Intensity Function* which describes the distribution of helium atoms that reach the final polariser according to initial and final wavelengths. When combining the polarisation along the x and y-direction after (S6) to a complex quantity and by introducing the scaled variables (κ_i, κ_f) which have been defined in (9) in the main text the polarisation becomes:

$$P(\boldsymbol{\kappa}) = \int \rho(\boldsymbol{\lambda}) e^{2\pi\mathrm{i}\boldsymbol{\kappa}\cdot\boldsymbol{\lambda}}\,\mathrm{d}\boldsymbol{\lambda}, \qquad \boldsymbol{\lambda} = (\lambda_i,\lambda_f), \boldsymbol{\kappa} = (\kappa_i,\kappa_f) \in \mathbb{R}^2. \tag{S7}$$

which is the two-dimensional Fourier transform introduced in (10) of the main text.

S.4 The Fourier Slice Theorem

Since the wavelength intensity function is supported around the average initial wavelength it could be viewed as a one-dimensional function that has been smoothed out into two-dimensions by the spread of initial wavelengths. A reduction of the two-dimensional problem to a one-dimensional one is possible due to the Fourier slice theorem which has been introduced in the main text. Suppose we rotate the $\boldsymbol{\lambda} = (\lambda_i,\lambda_f)$ coordinate system by an angle α to a new system $\boldsymbol{\tau} = (\tau_1,\tau_2) = R_\alpha(\boldsymbol{\lambda})$. Then one can derive the formula

$$P_\alpha(\kappa_i) = P(R_\alpha(\kappa_i,0)) = P(\kappa_i\cos\alpha, -\kappa_i\sin\alpha) = \int\Big(\int \rho(R_\alpha(\tau_1,\tau_2))\,\mathrm{d}\tau_2\Big)\exp(2\pi\mathrm{i}\kappa_i\tau_1)\,\mathrm{d}\tau_1. \tag{S8}$$

Therefore if we let $\rho_\alpha(\tau_1)$ denote the integral of $\rho(\lambda)$ along the line $\{R_\alpha(\tau_1,\tau_2) : \tau_2 \in \mathbb{R}\}$ then we have

$$P(\kappa_i\cos\alpha, -\kappa_i\sin\alpha) = \int \rho_\alpha(\tau_1)\exp(2\pi\mathrm{i}\kappa_i\tau_1)\,\mathrm{d}\tau_1. \tag{S9}$$

This is (12) of the main text, which was introduced as the Fourier slice theorem. It says that the restriction of P along the line $\{(\kappa\cos\alpha, -\kappa\sin\alpha),\ \kappa \in \mathbb{R}\}$, corresponds to the Fourier transform of ρ_α.

As mentioned in the main text, an elastic peak lies along the line $\lambda_i = \lambda_f$ and an integration angle of $\alpha = \pi/4$ produces the best results for resolving this feature as a single spike. Since the spread of initial wavelengths is already rather concentrated, the Fourier slice theorem is often used to treat this distribution as a single point. This point, the average wavelength λ_{av}, is either known beforehand or deduced by projecting along the λ_f-axis. With the incident wavelength $\lambda_i = \lambda_{\text{av}}$ fixed, any projection can be converted to a function in terms of λ_f only:[S6]

$$\lambda_f = \lambda_{\text{av}}\cot\alpha - \lambda_{\text{proj}}\sec\alpha, \tag{S10}$$

where $\lambda_{\text{proj}} \in \mathbb{R}$ is a point on the line to which we project.

S.5 Molecular Diffusion

In this section we shall focus on the description of the theoretical framework on which surface diffusion relies. If one wants to determine how effective a surface behaves, e.g. for catalysis, then one needs to study how molecules move on top of the surface over time. This is very different to the previous phonon examples covered earlier because we are no longer just considering the motions of nuclei in the lattice. Instead, we have species (molecules, atoms) covering large distances on surfaces while they interact with each other and with the substrate.

An important question is how one can differentiate between different types of diffusive regimes on a surface based on experimental measurements.

Scattering Cross Sections and the Van Hove Formalism The Van Hove formalism was initially developed for thermal neutron scattering.[S7] It provides us with a very powerful formalism to understand experimental neutron scattering spectra in terms of diffusive regimes:[S8] It establishes the relation between experimental functions such as the intermediate scattering function, the scattering function and the generalized correlation function in time and space, also known as *Van Hove correlation function* $G(\mathbf{r},t)$. The latter is defined as the probability of finding a particle at a certain position $\mathbf{r}$ and at a certain time t, provided the very same particle (in the case of self-diffusion) or a different one (distinct diffusion) was at the origin $\mathbf{r} = \mathbf{0}$ at time $t = 0$. In this paragraph we shall mention a few key results from this theory. This theoretical approach makes some assumptions that generally hold well for neutrons, for example

- The incoming beam of neutrons has a fixed incident wavevector $\mathbf{k}_i$ (and therefore a fixed incident energy). This is equivalent to monochromatisation of the beam.
- The potential for scattering of each nuclei is modelled as a Fermi pseudopotential, which is a delta spike at its position. This does *not* hold well for ^{3}He.
- A scattered neutron interacts with the bulk potential at most once. Although this assumption neglects the possibility of multiple scattering events it simplifies the theoretical framework considerably.[S8]

With these assumptions one can compute the differential scattering cross section $\frac{\mathrm{d}^2\sigma}{\mathrm{d}\Omega \mathrm{d}(\hbar\omega)}$, defined as the number of neutrons scattered into a solid angle $\mathrm{d}\Omega$ with change of energy $\mathrm{d}(\hbar\omega)$ divided by the flux of incident neutrons [S7, equation 4.13]:

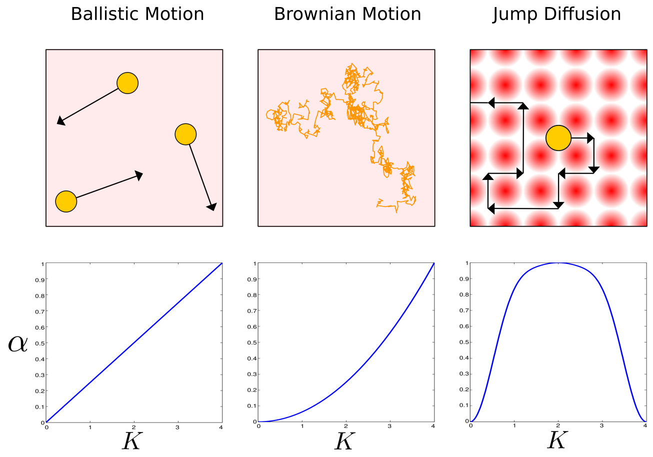

Figure S3. A depiction of three diffusive regimes. Orange denotes molecules on the surface and red denotes the surface potential. In the jump diffusion case the molecules are assumed to move instantly between vacant sites where the potential energy is smallest. The corresponding dephasing rates are shown in the lower plots.

$$\left(\frac{\mathrm{d}^2\sigma}{\mathrm{d}\Omega d(\hbar\omega)}\right) = \frac{N\sigma}{4\pi}\frac{k_f}{k_i}S(\mathbf{q},\omega), \tag{S11}$$

where N is the total number of nuclei, σ is the total scattering cross section, $k_i = |\mathbf{k}_i|, k_f = |\mathbf{k}_f|$ are the magnitudes of the incoming and outgoing scattering wavevectors, $\mathbf{q} = \mathbf{k}_i - \mathbf{k}_f$, $\hbar\omega$ denotes the change in energy (from $E = \hbar\omega$) and $S(\mathbf{q},\omega)$ is called the *Scattering Function* (SF). What makes (S11) particularly useful is that the SF can be related to the motion of nuclei in the bulk via multiple Fourier transforms:

$$\begin{aligned} G(\mathbf{r},t) &= \int_{\mathbb{R}^3} \exp(-2\pi \mathrm{i}\mathbf{q}\cdot\mathbf{r}) I(\mathbf{q},t)\,\mathrm{d}\mathbf{q} \\ &= \int_{\mathbb{R}^3}\int_{\mathbb{R}} \exp(-2\pi \mathrm{i}(\mathbf{q}\cdot\mathbf{r}+\omega t)) S(\mathbf{q},\omega)\,\mathrm{d}t\,\mathrm{d}\mathbf{q}. \end{aligned} \tag{S12}$$

The function $G(\mathbf{r},t)$ is often called the *Van Hove correlation function* and $I(\mathbf{q},t)$ the *Intermediate Scattering Function* (ISF). The correlation function $G(\mathbf{r},t)$ contains information about both self- and collective diffusion. However, if the spatial correlations between particles are negligible, the scattering function is essentially sensitive to the dynamics of a single particles (self-diffusion).

S.5.1 Extension to HeSE

Assuming that there is no correlation between particle dynamics, formula (S11) can be adapted[S6] to the surface sensitive helium scattering approach using

$$\frac{\mathrm{d}^2\sigma}{\mathrm{d}\Omega\mathrm{d}\omega} = S(\mathbf{K},\omega)\cdot|F(\mathbf{K},\omega)|^2. \tag{S13}$$

Instead of $\mathbf{q}$ used in (S11), $\mathbf{K}$ is typically used to denote to the momentum transfer. Due to the scattering geometry $\mathbf{K}$ now refers to $\mathbf{k}_f - \mathbf{k}_i$ projected onto the surface (like in (S1)) and $F(\mathbf{K},\omega)$ denotes the *Form Factor* that takes into account the fact that ^{3}He atoms are scattered from the electronic cloud of the atoms/molecules on the surface. This contrasts with the nuclear scattering of neutrons. For simplicity, we shall be assuming that the form factor can be compensated for when interpreting the scattering data, which effectively means setting this term to be equal to 1. Similarly (S12) still holds but for the two-dimensional equivalents of $G(\mathbf{r},t), I(\mathbf{q},t)$ and $S(\mathbf{q},t)$ (with $\mathbf{q}\in\mathbb{R}^3$ replaced by $\mathbf{K}\in\mathbb{R}^2$ and $\mathbf{R}\in\mathbb{R}^2$).

The signature of different diffusive regimes is contained in the dependence of the dephasing rate of the ISF or the quasi-elastic broadening of the SF on the momentum transfer.[S8] Examples for the fingerprint of different self-diffusive regimes are presented in Fig. S3. For the Brownian/ballistic/jump motion the dephasing rate shows a quadratic/linear/periodic dependence upon $\mathbf{K}$.[S6]

S.6 Changing Variables: From the Wavelength Intensity Function to the Scattering Function

As mentioned in the main text, the Van Hove correlation function cannot be directly measured. Nevertheless its properties can be inferred from partial knowledge of the experimental determined SF/ISF which consequently contains information about the diffusive processes. Hence we need to connect up the dots between the Van Hove formalism described in the previous section S.5 and the experimental background.

Recall that by (S13) we know that the scattering cross section is expressed in terms of change of surface momentum $\mathbf{K}$ and energy $E=\hbar\omega$. Therefore if one wants to convert the wavelength intensity function $\rho(\lambda,\theta_i)$ to the SF $S(\mathbf{K},\omega)$, i.e. to intensity in terms of energy or wavelength, one first needs to change variables (recalling (S1) and (S2)):

$$\begin{aligned}\omega &= \frac{E}{\hbar} = \frac{\hbar}{2m}k_f^2 - \frac{\hbar}{2m}k_i^2 = \frac{\pi h}{m}\left(\frac{1}{\lambda_f^2}-\frac{1}{\lambda_i^2}\right)\\ K &= k_f\sin\theta_i - k_i\sin\theta_f = \frac{2\pi}{\lambda_f}\sin\theta_i - \frac{2\pi}{\lambda_i}\sin(\theta_{SD}-\theta_i).\end{aligned} \tag{S14}$$

Here $\theta_{SD}=\theta_i+\theta_f$ is the total scattering angle between the source/surface/detector setup which cannot be changed for the Cambridge spin-echo apparatus. Instead one tilts the surface in order to vary the incident angle θ_i, which in turn determines θ_f. The direction of the change in surface momentum $\mathbf{K}$ is determined by the geometry of the apparatus but is always parallel to the surface (formally when we restrict $\mathbf{K}$ to the plane in the source/surface/detector setup it becomes a scalar hence why we only have a scalar K in (S14)).

From (S14) we see that the initial scattering angle θ_i is also an important variable in our experiments, therefore it is convenient to explicitly declare this dependency by rewriting $\rho(\lambda_i,\lambda_f)$ as $\rho(\lambda_i,\lambda_f,\theta_i)$. Our goal is to convert knowledge of $\rho(\lambda_i,\lambda_f,\theta_i)$ to knowledge of the scattering cross section/SF and then to the ISF. In this paper we focus on the approach where we fix λ_i (using a Fourier slice) and θ_i, leaving a function of one variable $\rho(\lambda_f)$, like in the phonon case.

By fixing λ_i,θ_i we only know $\tilde{S}(K,\omega)$ on a one-dimensional path in (K,ω) space. Because of this issue, some choose to exclusively work with the energy ω. With this approach the wavelength intensity function is converted into a frequency intensity function, which we interpret as $S(\omega)$. This involves using the change of variables (S14) along with a Jacobian term to preserve intensity:

$$S(\omega(\lambda_f)) = \rho(\lambda_f)\cdot\left(\frac{\mathrm{d}\omega}{\mathrm{d}\lambda_f}(\lambda_f)\right)^{-1}. \tag{S15}$$

From here one can Fourier transform $S(\omega)$ to derive an approximation to the ISF I in (S12) as in Ref.[S6] One problem however, is that the paths taken in (K,ω) space, the so-called *scan curves*, with θ_i,λ_f fixed are not straight lines parallel to the K-axis which otherwise would have justified this method using the Fourier slice theorem. Because of this issue we refer to $I(t)$ as an *approximate-ISF*.

S.7 Experimental Details

S.7.1 Sample Preparation

The single crystals used in the study were discs with a diameter of 10 mm and a thickness of 1 mm. The crystals were mounted on the sample holder which can be heated using a radiative heating from a filament on the backside of the crystal or cooled down to 100 K using liquid nitrogen. The sample temperature was measured using a chromel-alumel thermocouple.

Prior to the measurements the surface was cleaned by several Ar^+ sputtering and annealing cycles. For the Au(111) surface this included cycles of sputtering with 0.5 kV Ar^+ ions, 5 μA current for 15 minutes followed by annealing to 800 K (1 min). Ag(001) was typically sputtered with 0.8 kV Ar^+ ions, 8 μA current for 20 minutes, followed again by annealing to 800 K (2 min). The base pressure in the scattering chamber was $< 3 \cdot 10^{-11}$ mbar and the surface quality was monitored using helium reflectivity measurements.

The Au(111) experiments were performed at a sample temperature of $T_S = 200$ K, at which the surface remained clean, with no measured decrease in the reflectivity, for a period of at least four hours. After this time any adsorbed contaminants were removed by flashing the crystal to 500 K before continuing measurements. The Ag(001) experiments were done with the crystal above room temperature where the surface remains clean for several days.

For the deposition of cobalt phthalocyanine (CoPc, $C_{32}H_{16}CoN_8$) on Ag(001), a home-built Knudsen cell was used where a crucible filled with CoPc is resistively heated. The Knudsen cell was mounted in a separate dosing arm and was inserted into the scattering chamber for deposition of CoPc onto the clean Ag(001) surface. CoPc was deposited at a surface temperature of 350 K and a typical dosing pressure of $2 \cdot 10^{-9}$ mbar until the elastically scattered He signal had been attenuated by a factor of 2.

S.7.2 Experimental Parameters

The experimental parameters for the measurements presented in this study are summarised in table S1.

Table S1. Experimental parameters for the measured systems presented in this work.

Measured system	Au(111)	CoPc/Ag(001)
Sample temperature	200 K	350 K
Incident He energy E_i	8.0 meV	8.1 meV
Current range	[-4,4] A	[0,10] A
Number of sampled points	2048	101
Incident angle θ_i	19.325°	24.2°
Momentum transfer ΔK (for elastic scattering)	$0.32\ \text{Å}^{-1}$	$-0.22\ \text{Å}^{-1}$

References

[S1] Hofmann, P. *Surface Physics: An Introduction* (eBook 978-87-996090-1-7, 2013).

[S2] Kittel, C. *Introduction to Solid State Physics* (John Wiley & Sons Inc, 2005).

[S3] Benedek, G. *et al.* Theory of surface phonons at metal surfaces: recent advances. *J. Phys.: Condens. Matter* **22**, 084020 (2010).

[S4] Kress, W., de Wette & W., F. *Surface Phonons*. No. 21 in Springer Series in Surface Sciences (Springer Berlin Heidelberg, 1991).

[S5] Heid, R. & Bohnen, K. P. Ab initio lattice dynamics of metal surfaces. *Phys. Rep.* **387**, 151–213 (2003).

[S6] Jardine, A. P., Hedgeland, H., Alexandrowicz, G., Allison, W. & Ellis, J. Helium-3 spin-echo: Principles and application to dynamics at surfaces. *Prog. Surf. Sci.* **84**, 323–379 (2009).

[S7] Squires, G. L. *Introduction to the Theory of Thermal Neutron Scattering* (Cambirdge University Press, 1978).

[S8] Bée, M. *Quasielastic Neutron Scattering* (CRC Press, 1988).
